# Boiogito, a Kampo medicine, improves hydrarthrosis in a rat model of knee osteoarthritis

**DOI:** 10.1186/s12906-015-0979-7

**Published:** 2015-12-24

**Authors:** Naoki Fujitsuka, Mitsuo Tamai, Kazuaki Tsuchiya, Seiichi Iizuka, Naoko Tsuchiya, Bunsho Makino, Tomohisa Hattori, Yoshio Kase, Yoichiro Isohama

**Affiliations:** Tsumura Research Laboratories, Tsumura & Co., 3586 Yoshiwara, Ami-machi, Inashiki-gun, Ibaraki 300-1192 Japan; Botanical Raw Materials Division, Botanical Raw Materials Research Laboratories, Tsumura & Co., Ami-machi, Ibaraki 300-1192 Japan; Laboratory of Applied Pharmacology, Faculty of Pharmaceutical Sciences, Tokyo University of Sciences, Chiba, 278-8510 Japan

**Keywords:** Aquaporin, Boiogito, Hyaluronan, Osteoarthritis, Synovial fluid

## Abstract

**Background:**

Hydrarthrosis, which is associated with knee pain and limited range of motion, decreases the quality of life (QOL) of patients with osteoarthritis (OA). The Kampo medicine boiogito is prescribed for the treatment of knee OA with hydrarthrosis; however, its precise mechanisms of action remain unknown. The purposes of this study were to assess the pharmacological effects of boiogito and its mechanisms of action on joint effusion in rats with surgically induced OA.

**Methods:**

A rat OA model was produced by transecting the anterior (cranial) cruciate ligament, medial collateral ligament, and medial meniscus in the right knee joints of 7-week-old female Wistar rats. The rats were given chow containing boiogito (1 or 2 %) or indomethacin (0.002 %) for 4 weeks after surgical transection. Levels of interleukin-1β (IL-1β) and hyaluronic acid (HA) were measured by enzyme-linked immunosorbent assay. Knee joint pain was assessed using an incapacitance tester. Osmotic water permeability in cultured rabbit synovial cells was assessed using stopped-flow analysis.

**Results:**

Increased synovial fluid volume and knee joint pain were observed in rats with surgically induced OA. In rats with OA, levels of IL-1β and HA in the articular cavity were higher but concentration of HA in synovial fluid was lower than in sham-operated rats, suggesting excessive synovial fluid secretion. Administration of boiogito improved hydrarthrosis, IL-1β, and HA concentrations and alleviated knee joint pain in rats with OA. Indomethacin reduced IL-1β and knee joint pain but failed to improve hydrarthrosis or HA concentration in rats with OA. Osmotic water permeability in synovial cells, which is related to the function of the water channel aquaporin, was decreased by treatment with boiogito.

**Conclusion:**

Boiogito ameliorates the increased knee joint effusion in rats with OA by suppressing pro-inflammatory cytokine IL-1β production in the articular cavity and regulating function of water transport in the synovium. The improvement of hydrarthrosis by boiogito results in the increased HA concentration in synovial fluid, thus reducing joint pain. Boiogito may be a clinically useful treatment of QOL in patients with OA with hydrarthrosis.

**Electronic supplementary material:**

The online version of this article (doi:10.1186/s12906-015-0979-7) contains supplementary material, which is available to authorized users.

## Background

Osteoarthritis, which is characterized by inflammation and degradation of the cartilage matrix, is triggered mainly by excessive joint forces and leads to pathological changes in the joints. Knee OA is a common problem in older women who complain of joint pain [1]. Nonsteroidal anti-inflammatory drugs are widely used as an effective and conservative treatment for knee OA. However, serious problems such as gastrointestinal tract adverse events result from long-term NSAID use [2]. Meanwhile, the intra-articular injection of HA, especially high-molecular-weight HA, has been shown to maintain the cartilage matrix and minimize inflammation by lubricating and cushioning joints. However, its use carries the risk of local adverse events including effusions or flares [3, 4].

Hydrarthrosis is associated with pain and limitation in the range of knee motion; these are critical clinical symptoms of OA. It is caused predominantly by synovial inflammation, which is mediated by cytokines such as IL-1β [5]. In addition, AQP is a water channel and maintains body water homeostasis. Some AQP isoform expressions are increased in the articular joints of patients with OA and rheumatoid arthritis, suggesting the relevance of hydrarthrosis [6, 7]. Synovial fluid aspiration is commonly performed as a palliative therapy for hydrarthrosis.

Boiogito, a traditional Japanese herbal (Kampo) medicine, is prescribed as a remedy for arthritis, nephrosis, edema, hyperhidrosis, and obesity. In particular, it is effective for these diseases in patients with the symptoms of fatigue, light-complexion, and soft-muscle. The usual adult dose is 7.5 g/day orally divided over two or three doses before or between meals. The dosage may be adjusted according to the patient’s age and body weight and symptoms [8]. Boiogito is an extract composed of six herbal drugs included Sinomenium Stem and Astragalus Root. Sinomenine, a ingredient extracted from the Sinomenium Stem, has demonstrated potential anti-inflammatory activity. In vitro studies have shown that sinomenine inhibit lymphocyte proliferation [9, 10] and decreased eicosanoid synthesis and nitric oxide production of macrophages [11]. In rat adjuvant- and collagen-induced arthritis, sinomenine treatment improved arthritic score and hind paw swelling [12]. Astragalus Root also has anti-inflammatory activity through decreasing production of cytokine, Interferon-γ or Tumor necrosis factor-α in a rat model of autoimmune myocarditis, in NC/Nga mice and in db/db diabetic mice [13–15]. These reports suggest boiogito have also possibility for decreasing inflammation such as osteoarthritis. A recent clinical study [16] demonstrated that oral administration of boiogito is a possible treatment for knee joint effusion in OA patients and has no severe adverse effects; however, its mechanisms remain unknown. In this study, we examined the effect of boiogito on hydrarthrosis and its mechanism of action using a rat model of surgically induced OA and cultured synovial cells.

## Methods

### Surgically induced OA animal model

Female Wistar rats were purchased from Japan Charles River Laboratory (Tokyo, Japan) and housed in a regulated environment. Standard laboratory chow and water were available ad libitum. All experimental procedures were performed according to the “Guidelines for the Care and Use of Laboratory Animals” and approved by the Laboratory Animal Committee of Tsumura & Co. (Tokyo, Japan).

Seven-week-old rats were anesthetized with intraperitoneal injections of sodium pentobarbital, and surgical sites were shaved. The right knee joint of each rat was exposed; the medial collateral and anterior (cranial) cruciate ligaments were transected and the medial meniscus was resected using a microsurgical knife. In the sham-operated rats (*n* = 8), the right knee joint was exposed and then closed.

### Drug treatment

**Fig. 1:**
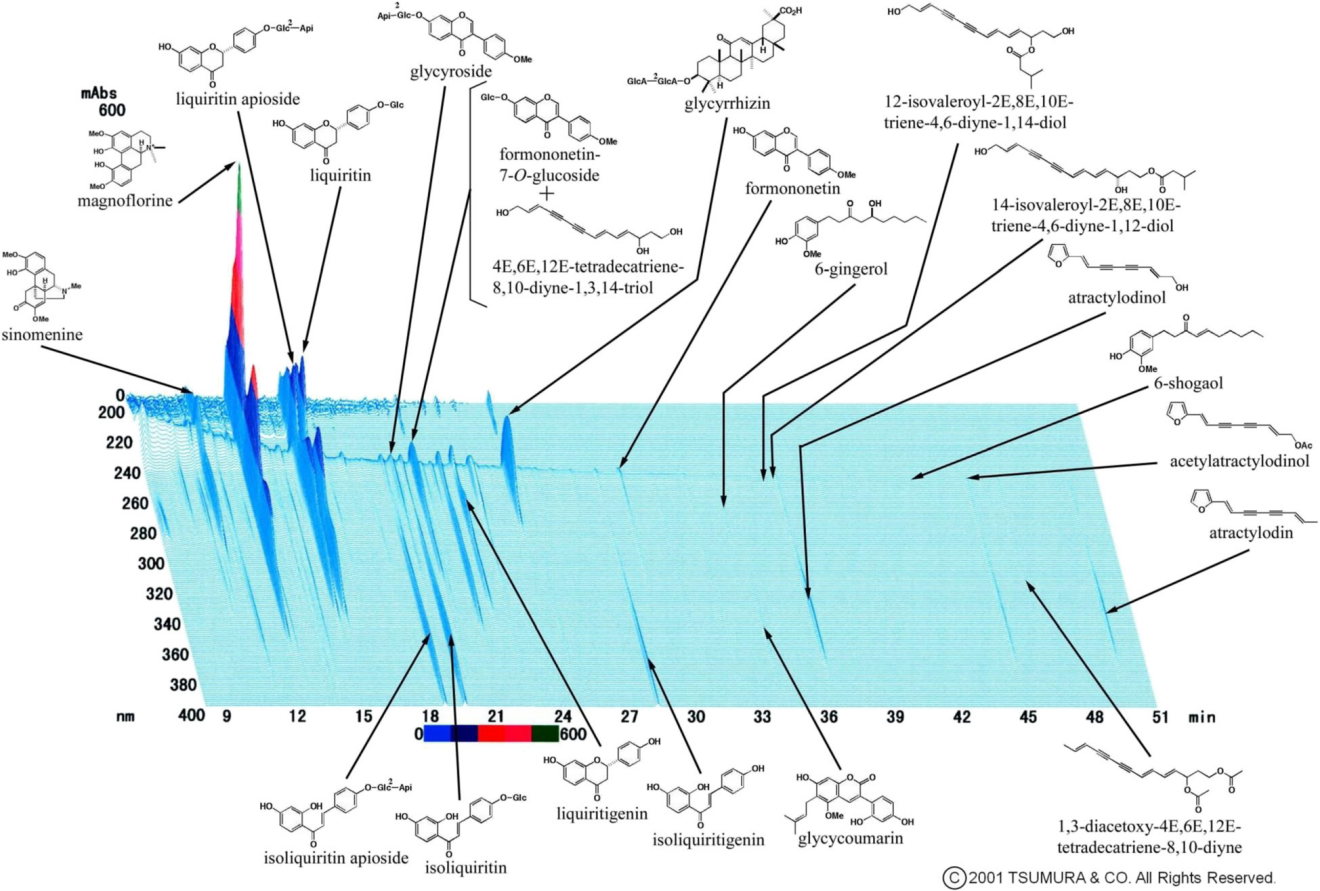
A three-dimensional high-performance liquid chromatography profile of boiogito, provided by Tsumura & Co

The Kampo medicine boiogito was obtained by Tsumura & Co. (Tokyo, Japan). Boiogito is a powdered extract produced under stringent manufacturing practices by spray drying a hot-water extract of the herbal mixture, which is composed of the following six Japanese Pharmacopoeia standard herbal constituents in fixed proportions: Sinomenium Stem (*Sinomeni Caulis et Rhizoma*) 5.0 g/18.5 g, Astragalus Root (*Astragali Radix*) 5.0 g/18.5 g, Atractylodes Lancea Rhizoma (*Atractylodis Lanceae Rhizoma*) 3.0 g/18.5 g, Jujube (*Zizyphi Fructus*) 3.0 g/18.5 g, Glycyrrhiza (*Glycyrrhizae Radix*) 1.5 g/18.5 g, and Ginger (*Zingiberis Rhizoma*) 1.0 g/18.5 g. Kampo medicines, including boiogito, are standardized with respect to the quality and quantity of ingredients and are prescribed for the treatment of several diseases under the oversight of the Japanese Ministry of Health, Labour and Welfare. A three-dimensional high-performance liquid chromatography profile of boiogito provided by Tsumura & Co. is shown in Fig. 1. Numerous major ingredients included in boiogito are identified.

The rats with OA were given chow containing boiogito (1 or 2 %, *n* = 12), indomethacin (0.002 %, *n* = 11; Sigma, St. Louis, MO, USA), or control chow (*n* = 12). The mean daily doses of 1 and 2 % boiogito and indomethacin were 0.73 g/kg, 1.49 g/kg, and 1.48 mg/kg, respectively. The assays and tests described below were conducted 4 weeks after surgery.

### Incapacitance test

Weight-bearing changes in the rats with OA were measured using an incapacitance tester [17]. Postural imbalance, which reportedly indicates a change in the pain threshold and weight distribution of the limbs, was decreased. Each rat stood on its hind limbs on the incapacitance apparatus, and the weight borne by each hind limb was measured for 5 s. The ratio of the weight borne by the right to left hind limb was calculated. The mean of 10 consecutive measurements for each rat was recorded.

### Analytical assays

After the rats were sacrificed, synovial fluid in the articular cavity was collected using filter paper, and fluid volume was estimated by weight. The synovial fluid samples were eluted from the filter paper by immersion in 250 μL of stirred ice-cold PBS for 10 min. The levels of HA (Hyaluronan Assay Kit; Seikagaku Co., Tokyo, Japan) and IL-1β (Bio-Plex; Bio-Rad, Hercules, CA, USA) in the synovial fluid samples were measured by enzyme-linked immunosorbent assay. HA concentration was calculated by dividing HA level by fluid volume.

### Gene expression assay

Synovial membrane gene expression levels were measured using a real-time polymerase chain reaction system (ABI 7900HT; Applied Biosystems, CA, USA). Total RNA was extracted from the synovial membrane samples, which were collected after the rats were sacrificed, using an RNeasy Mini Kit, and DNA was removed from RNA using RNase-Free DNase (Qiagen, Valencia, CA, USA). Reverse transcription reactions were performed using a TaqMan reverse transcription kit (Applied Biosystems). All oligonucleotide primers and fluorogenic probe sets for TaqMan real-time PCR were obtained from Applied Biosystems; these included matrix metalloprotease 3 (*MMP3*; Rn00591740_m1), hyaluronan synthase 2 (*HAS2*; Rn00565774_m1), hyaluronidase 1 (*HYAL1*; Rn02133715_s1), and aquaporin 1 (*AQP1*; Rn00562834_m1). Beta actin (*ACTB*; Rn00667869_m1) was used as an endogenous control.

### Histochemical study

Articular cartilage was collected after the rats were sacrificed. It was fixed in 15 % phosphate-buffered formalin, decalcified and embedded in paraffin. Histologic sections 4 μm in thickness were stained with Safranin O for light microscopic examination. Modified Mankin scores were obtained using a semi-quantitative pathological scoring system: Structure (0, normal; 1, surface irregularities; 2, pannus and surface irregularities; 3, clefts within the transitional zone; 4, clefts within the radial zone; 5, clefts within the calcified zone; and 6, complete disorganization), Cell (0, normal; 1, diffuse hypercellularity; 2, cloning; and 3, hypocellularity), and Safranin O staining (0, normal; 1, slight reduction; 2, moderate reduction; 3, severe reduction; and 4, no dye noted).

### Osmotic water permeability in synovial cells

Osmotic water permeability was assessed using a stopped-flow analysis [18]. Rabbit synovial cells (HIG-82 [ATCC CRL-1832]; ATCC, Manassas, VA, USA) were cultured in 10 % fetal bovine serum in Hams’ F12 medium. Cells were incubated in the medium for 90–100 min after detachment with trypsin/EDTA and placed in ice-cold PBS. The cell suspensions (10^6^ cells/mL) were rapidly mixed with equal volumes of 600 mmol/L mannitol-containing PBS at 10 °C using a stopped-flow apparatus (SX18MV-R; Applied Photophysics Ltd., Leatherhead, Surrey, UK). They were then illuminated by a 400-nm light, and the light scattering was measured for 5 s. The scattered light intensity was fit to a single exponential curve of 1.5 s, and the osmotic water permeability was calculated using the following equation:

$$ \mathrm{P}\mathrm{f}\left(\mathrm{cm}/\mathrm{s}\right)=\left[\mathrm{d}\left(\mathrm{V}/\mathrm{V}0\right)/\mathrm{d}\mathrm{t}\right]/\left[\mathrm{V}\mathrm{w}\times \mathrm{S}\mathrm{A}\mathrm{V}\times \mathrm{dOSM}\right], $$

where Pf is the osmotic water permeability, d(V/V0)/dt is the initial curve slope, Vw (cm^3^/mol) is the molar ratio of water, SAV (cm^-1^) is the surface area to the initial cell volume, and dOSM (mol/cm^3^) is the osmolality of the extracellular solution.

Boiogito and the extracts of its six constituents were dissolved in a solution of 5 % dimethylsulfoxide in PBS and centrifuged for 5 min at 10,000 rpm. The supernatants (0.1 mL) were added to the cell suspension and 600 mmol/L of mannitol-containing PBS (final volume, 1 mL). Osmotic water permeability activity was examined 15 min after the reaction. The AQP inhibitor mercuric chloride was assayed as a positive control.

### Statistical analysis

Values for individual groups are shown as mean ± standard error (SE). To assess differences among groups, the Student *t*-test, a multi-group Dunnett test, or the Steel test was performed. Values of *P* < 0.05 were considered statistically significant.

## Results

### Surgically induced OA model

**Fig. 2:**
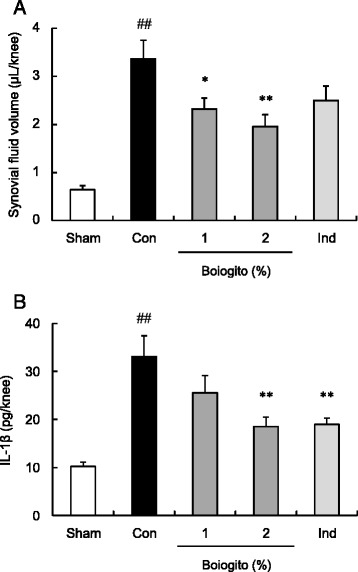
Effects of boiogito and indomethacin on hydrarthrosis in a rat model of knee osteoarthritis (OA). Rats with OA were given chow containing boiogito (1 or 2 %, *n* = 12) or indomethacin (Ind; 0.002 %, *n* = 11) for 4 weeks. Increased synovial fluid volume and interleukin-1β in the articular cavity were observed in rats with surgically induced OA. **a**: Synovial fluid volume in rats with OA was decreased by boiogito. **b**: The level of interleukin-1β in the articular cavity in rats with OA was reduced by boiogito and Ind. Results are expressed as mean ± standard error (SE). ^##^ *P* < 0.01 vs. sham-operated rats (Sham, *n* = 8), *P < 0.05, **P < 0.01 vs. non-treated control rats with OA (Con, *n* = 12)

Increased synovial fluid volume was observed in rats with OA. Daily administration of boiogito, but not indomethacin, significantly decreased the synovial fluid volume in rats with OA (Fig. 2a). IL-1β levels in the articular cavity were approximately three times higher in rats with OA than in sham-operated rats. Both boiogito and indomethacin were associated with decreased IL-1β levels in rats with OA (Fig. 2b).

**Fig. 3:**
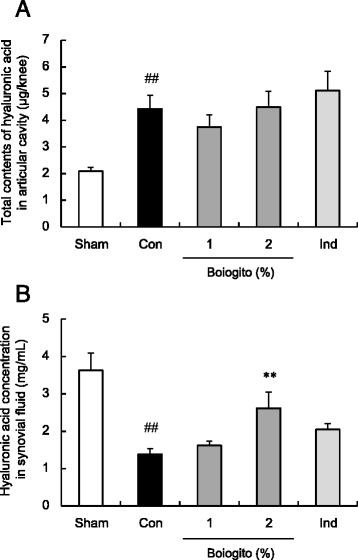
Effects of boiogito and indomethacin on hyaluronic acid (HA) in a rat model of knee osteoarthritis (OA). The contents of HA in the articular cavity (**a**) were increased but HA concentrations in the synovial fluid (**b**) were decreased in rats with OA (Con, *n* = 12) compared with those in sham-operated rats (Sham, *n* = 8). Daily administration of boiogito (2 %, *n* = 12) did not change the HA content but recovered the decreased HA concentration in synovial fluid. These parameters were not influenced by indomethacin (Ind, *n* = 11). Results are expressed as mean ± SE. ^##^ *P* < 0.01 vs. Sham, **P < 0.01 vs. non-treated control rats with OA (Con)

In rats with OA, the total content of HA in the articular cavity was increased (Fig. 3a) but the concentration of HA in the synovial fluid was decreased (Fig. 3b) compared with measurements in sham-operated rats. After daily administration of boiogito, there was no change in the HA content in the articular cavity, but the HA concentration in the synovial fluid had increased.

**Fig. 4:**
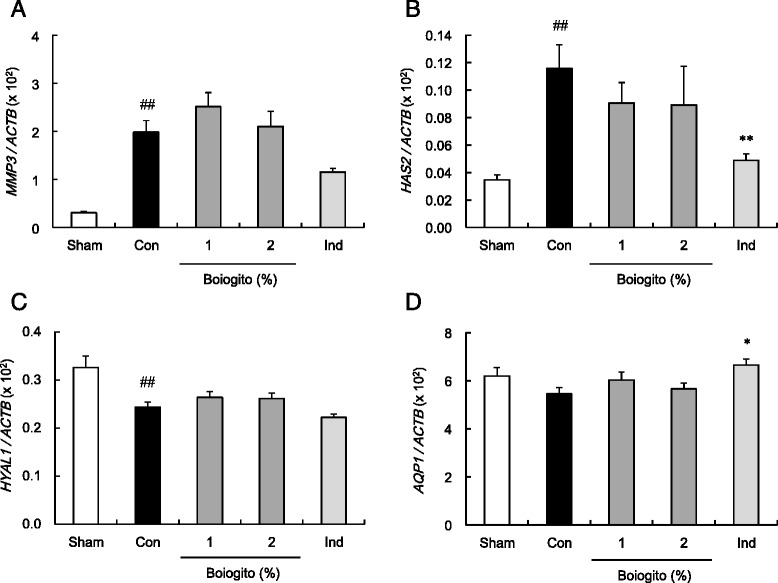
Effects of boiogito and indomethacin on gene expressions in the synovial membranes in a rat model of knee osteoarthritis (OA). Data are shown as the relative mRNA expression of matrix metalloprotease 3 (*MMP3*; **a**), hyaluronan synthase 2 (*HAS2*; **b**), hyaluronidase 1 (*HYAL1*; **c**) and aquaporin 1 (*AQP1*; **d**), normalized to reference gene of beta actin (*ACTB*). The expressions of *MMP3* (**a**) and *HAS2* (**b**) increased and that of *HYAL1* (**c**) decreased in the synovial membranes of rats with OA (Con, *n* = 12) compared with those of sham-operated rats (Sham, *n* = 8). Expressions of these genes were not influenced by boiogito (*n* = 12). Indomethacin (Ind, *n* = 11) decreased *HAS2* mRNA and slightly increased *AQP1* mRNA in the synovial membranes of rats with OA. Results are expressed as mean ± SE. ^##^ *P* < 0.01 vs. Sham, *P < 0.05, **P < 0.01 vs. non-treated control rats with OA (Con)

The gene expressions of *MMP3* (Fig. 4a) and *HAS2* (Fig. 4b) increased in the synovial membranes of rats with OA, whereas that of *HYAL1* (Fig. 4c) decreased. *AQP1* expressions (Fig. 4d) were not significantly different between rats with OA and sham-operated rats. Expressions of these genes were not influenced by boiogito. Indomethacin administration inhibited the increased *HAS2* expression and slightly increased the *AQP1* expression.

**Fig. 5:**
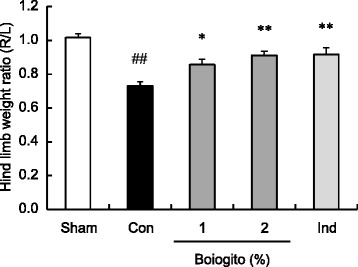
Effects of boiogito and indomethacin on joint pain in a rat model of knee osteoarthritis (OA). Joint pain was assessed by the weight ratio of the right (osteoarthritic) to left (contralateral control) hind limbs using an incapacitance tester. Rats with OA showed weight-bearing deficits associated with knee pain. Boiogito (*n* = 12) and indomethacin (*n* = 11) significantly alleviated weight-bearing deficits in rats with OA. Results are expressed as mean ± SE. ^##^ *P* < 0.01 vs. sham-operated rats (Sham, *n* = 8), *P < 0.05, **P < 0.01 vs. non-treated control rats with OA (Con, *n* = 12)

Rats with OA showed weight-bearing deficits, most likely caused by knee joint pain. Boiogito and indomethacin were associated with significantly improved weight-bearing ratios in rats with OA (Fig. 5).

Cartilage surface irregularities, diffuse hypercellularity, and decreased Safranin O staining were observed in joints of rats with OA 4 weeks after surgery. Daily administration of boiogito tended to alleviate the histochemical changes in articular cartilage of rats with OA (Additional file 1: Figure S1). Mankin score in OA rats decreased after treatment with boiogito, but not significantly (Control: 3.8 ± 0.4; Boiogito (1 %): 3.0 ± 0.9; Boiogito (2 %): 2.6 ± 0.9; Indomethacin: 3.1 ± 1.1).

### Osmotic water permeability in synovial cells

**Fig. 6:**
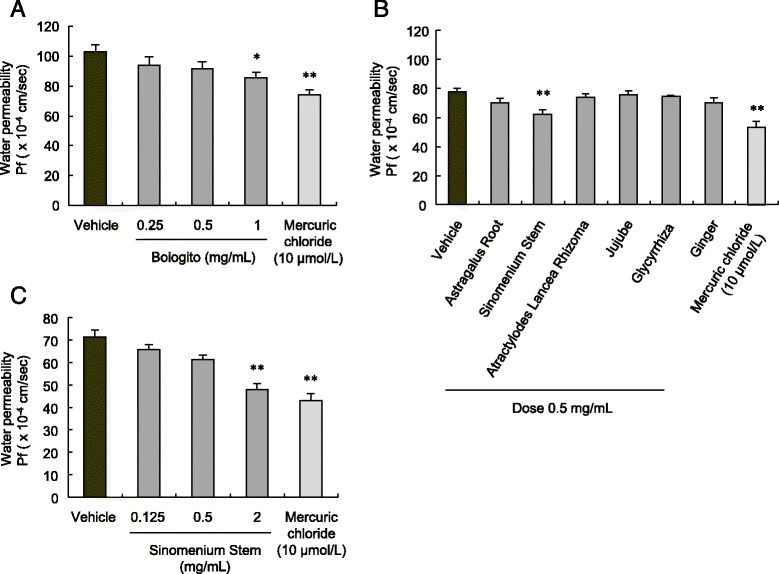
Effect of boiogito on water permeability in cultured synovial cells. **a** Boiogito (0.25–1 mg/mL) and the aquaporin inhibitor mercuric chloride (10 μmol/L) inhibited water permeability in synovial cells (*n* = 8–9). **b**, **c** Sinomenium Stem, one of six boiogito constituents, decreased water permeability of the synovial cells (*n* = 4–5) in a dose-dependent manner (*n* = 4–6). Results are expressed as mean ± SE. *P < 0.05, **P < 0.01 vs. vehicle

Boiogito (0.25–1 mg/mL) and the AQP inhibitor mercuric chloride (10 μmol/L) inhibited osmotic water permeability in the synovial cells (Fig. 6a). We examined the effect of the six boiogito constituents (0.5 mg/mL) and found that Sinomenium Stem showed a dose-dependent decrease in osmotic water permeability in the synovial cells (Fig. 6b, c).

## Discussion

We found that the Kampo medicine boiogito attenuated hydrarthrosis and joint pain and decreased IL-1β in the articular cavity in rats with surgically induced OA. Synovial tissue inflammation is known to be a pathogenic factor of OA of the knee. Several cytokines cause increased joint inflammation, particularly the pro-inflammatory cytokine IL-1β is most frequently detected in synovial tissues of OA patients [5]. Additionally, plasma levels of interleukin-1 receptor antagonist (IL1Ra) are modestly associated with the severity and progression of symptomatic knee OA [19]. Several reports have shown that IL-1β produces matrix metalloprotease, which degrade the cartilage matrix, in chondrocytes and synoviocytes [20–22]. We also demonstrated that intra-articular injections of IL-1β increased synovial fluid volume and the expression of *MMP3* in the synovial membrane in rats (data not shown). In this study, increased IL-1β in the articular cavity and expression of *MMP3* in the synovial membrane were observed in rats with OA. Boiogito decreased IL-1β, but not *MMP3*, in rats with OA. These results suggest that the inhibitory effect of boiogito on knee hydrarthrosis in rats with OA is mediated by the suppression of pro-inflammatory cytokine IL-1β production in the articular cavity. Sinomenine, a principal component of boiogito, has demonstrated potential anti-inflammatory activity [10]. In animal models of adjuvant- and collagen-induced arthritis, sinomenine improved symptoms and decreased tumor necrosis factor-α and IL-1β expressions. It was also shown to be involved in inhibiting nuclear factor-κB binding activity by upregulating IκB-α expression [12, 23]. Sinomenine may play a important role in improving hydrarthrosis in rats with OA in this study.

On the other hand, indomethacin decreased IL-1β in the articular cavity as well as boiogito but failed to inhibit joint effusion in rats with OA. These findings suggest that a decrease in IL-1β is necessary but insufficient for the improvement of hydrarthrosis. Water transport is a physiologically important system for maintaining body water homeostasis. AQPs are specific protein channels for water transport, and some AQP isoforms (AQP1, AQP3, and AQP9) are located in synovial microvessels, synoviocytes, and chondrocytes [24]. Immunohistochemistry revealed increased AQP1 expression in the articular joints of patients with rheumatoid arthritis, suggesting a potential role for synovial AQP1 in joint swelling, vasogenic synovial fluid formation, and hydrarthrosis associated with synovial inflammation [6, 25]. In the present study, the *AQP1* gene expression in the synovial membrane was not influenced by OA induction or boiogito treatment but slightly increased by indomethacin treatment. To assess the function of AQPs in synovial cells, we performed the stopped-flow analysis in vitro. The osmotic water permeability of the synovial cells was inhibited by boiogito and the AQP inhibitor mercuric chloride. These findings suggest that the inhibitory effect of boiogito on water transport in synovial cells, which seems to be mediated by AQPs, may be partially related to a decrease in joint effusion in rats with OA. Sinomenium Stem, one of the constituents of boiogito, also inhibited the osmotic water permeability of the synovial cells. However, the effect of Sinomenium Stem is insufficient to completely explain the effect of boiogito, which is possibly associated with the synergistic effect of other constituents. Future experiments will determine whether ingredients of boiogito have an AQP-mediated effect on hydrarthrosis.

HA in synovial fluid plays an important role in maintaining high fluid viscosity and preserving the normal cartilaginous matrix by lubricating and cushioning the joint [3]. A reduced concentration of HA is critical to cartilage disorder progression in OA. Decreased HA concentrations in synovial fluid were noted in rats with surgically induced OA. However, the total HA content of the articular cavity increased in rats with OA. This process is mediated by increased *HAS2* and decreased *HYAL1* in the synovial membrane, suggesting that HA production is accelerated in arthritis. Therefore, excessive hydrarthrosis appears to reduce HA concentrations in the synovial fluid of rats with OA. Daily administration of boiogito recovered the decreased HA concentration in the synovial fluid without changing the HA content of the articular cavity in rats with OA. These findings suggest that boiogito maintains the HA concentration by inhibiting joint effusion. The effect of boiogito on HA concentration appears to result in lubricating and cushioning the joint, thus reducing joint pain.

## Conclusions

Our study demonstrated that the Kampo medicine boiogito improves hydrarthrosis, resulting in an increased HA concentration in the synovial fluid and alleviation of knee joint pain in rats with surgically induced OA. Boiogito appears to alleviate hydrarthrosis by suppressing articular pro-inflammatory cytokine IL-1β production and inhibiting water transport through AQPs in the synovium. Boiogito may be a clinically useful treatment of frequent hydrarthrosis, which is associated with pain and limited range of motion in the knees of patients with OA.

## Additional file

Additional file 1: Figure S1. Histochemical changes in joints of rats with osteoarthritis. Cartilage surface irregularities, diffuse hypercellularity, and decreased Safranin O staining were seen in joints of rats with OA 4 weeks after surgery. Daily administration of boiogito and indomethacin partially tended to alleviate the histochemical changes in articular cartilage of rats with OA. (PDF 250 kb)

## Abbreviations

AQP: aquaporin
HA: hyaluronic acid
HAS2: hyaluronan synthase 2
HYAL1: hyaluronidase 1
IL-1β: interleukin-1β
MMP3: matrix metalloprotease 3
NSAID: nonsteroidal anti-inflammatory drug
OA: osteoarthritis
