# Supplementary material for: Boiogito, a Kampo medicine, improves hydrarthrosis in a rat model of knee osteoarthritis
Source: BMC Complement Altern Med. 2015 Dec 24;15:451. doi: 10.1186/s12906-015-0979-7 (PMC4691021; doi:10.1186/s12906-015-0979-7)
Supplement: Additional file 1: Figure S1. — Histochemical changes in joints of rats with osteoarthritis. Cartilage surface irregularities, diffuse hypercellularity, and decreased Safranin O staining were seen in joints of rats with OA 4 weeks after surgery. Daily administration of boiogito and indomethacin partially tended to alleviate the histochemical changes in articular cartilage of rats with OA. (PDF 250 kb) [file 12906_2015_979_MOESM1_ESM.pdf]

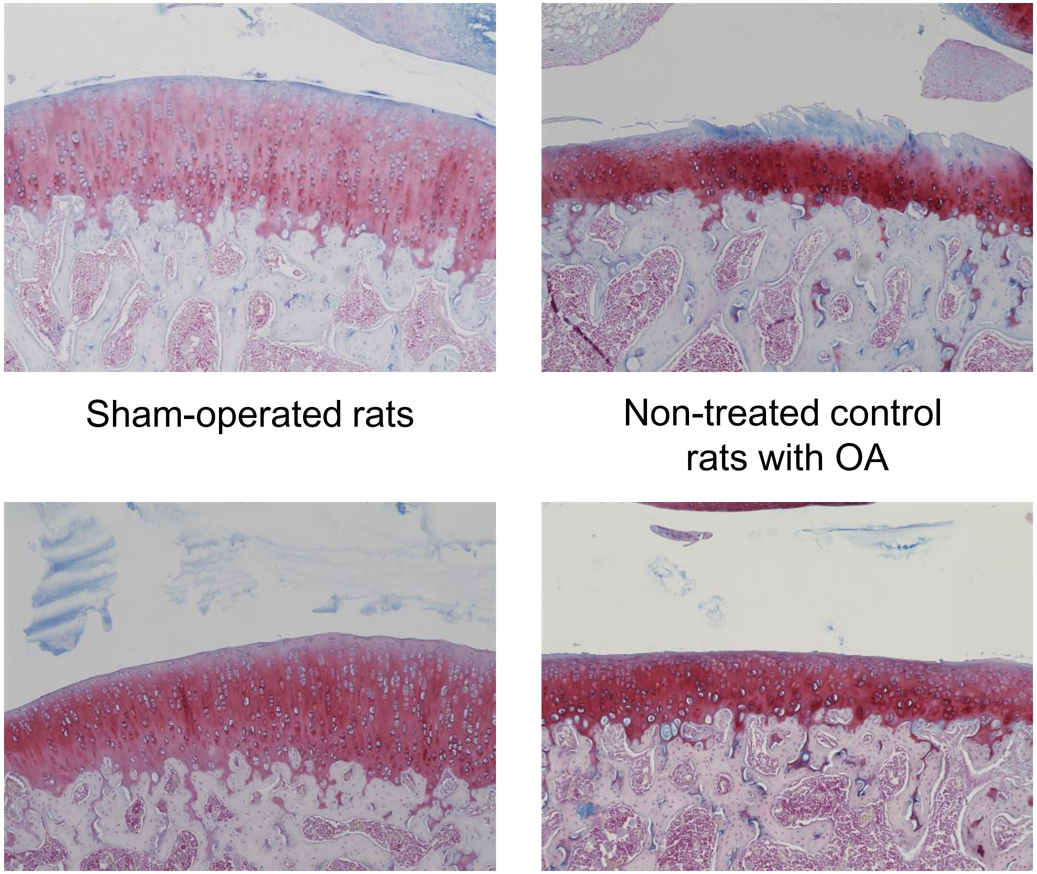

Sham-operated rats

Non-treated control rats with OA

Boiogito (2%)-treateed rats with OA

Indomethacin-treated rats with OA

Supplemental Fig. 1. Histochemical changes in joints of rats with osteoarthritis. Cartilage surface irregularities, diffuse hypercellularity, and decreased Safranin O staining were seen in joints of rats with OA 4 weeks after surgery. Daily administration of boiogito and indomethacin partially tended to alleviate the histochemical changes in articular cartilage of rats with OA.
